# First-Principles Study on the Adsorption and Dissociation of Impurities on Copper Current Collector in Electrolyte for Lithium-Ion Batteries

**DOI:** 10.3390/ma11071256

**Published:** 2018-07-21

**Authors:** Jian Chen, Chao Li, Jian Zhang, Cong Li, Jianlin Chen, Yanjie Ren

**Affiliations:** 1 School of Energy and Power Engineering, Changsha University of Science & Technology, Changsha 410114, Hunan, China; chenjian5130@163.com (J.C.); lichao460s@163.com (C.L.); zj4343@163.com (J.Z.); liconghntu@126.com (C.L.); cjlinhunu@csust.edu.cn (J.C.); 2 Key Laboratory of Energy Efficiency and Clean Utilization, Education Department of Hunan Province, Changsha University of Science & Technology, Changsha 410114, Hunan, China; 3 Guangxi Key Laboratory of Electrochemical Energy Materials, Guangxi University, Nanning 530004, Guangxi, China

**Keywords:** lithium-ion batteries, copper current collector, first-principles method, adsorption

## Abstract

The copper current collector is an important component for lithium-ion batteries and its stability in electrolyte impacts their performance. The decomposition of LiPF_6_ in the electrolyte of lithium-ion batteries produces the reactive PF_6_, which reacts with the residual water and generates HF. In this paper, the adsorption and dissociation of H_2_O, HF, and PF_5_ on the Cu(111) surface were studied using a first-principles method based on the density functional theory. The stable configurations of HF, H_2_O, and PF_5_ adsorbed on Cu(111) and the geometric parameters of the admolecules were confirmed after structure optimization. The results showed that PF_5_ can promote the dissociation reaction of HF. Meanwhile, PF_5_ also promoted the physical adsorption of H_2_O on the Cu(111) surface. The CuF_2_ molecule was identified by determining the bond length and the bond angle of the reaction product. The energy barriers of HF dissociation on clean and O-atom-preadsorbed Cu(111) surfaces revealed that the preadsorbed O atom can promote the dissociation of HF significantly.

## 1. Introduction

Since Armand et al. proposed the concept of rechargeable lithium rocking chair batteries in 1972, lithium-ion batteries have been used widely in portable electronic devices, electric cars, and the aerospace and military fields [1,2,3,4]. Although lithium-ion batteries exhibit excellent performance under ambient conditions, during cycling and storage their usable capacity decreases and internal resistance increases rapidly at elevated temperatures [5]. Accordingly, numerous attempts have been performed to increase the thermal stability of lithium-ion batteries. Some studies indicated that LiPF_6_, widely used as an electrolyte in lithium-ion batteries, is one of the important origins for the capacity fade at elevated temperatures. Ravdel et al. [6] analyzed the thermal decomposition of solid LiPF_6_ and found that LiPF_6_ thermally decomposes into PF_5_ and LiF. PF_5_ is a strong Lewis acid and easily reacts with the solid electrolyte interphase (SEI) in Li-ion batteries. Furthermore, the trace amount of water is always contained in electrolytes (≤50 ppm). Yang et al. [7] investigated the thermal stability of the neat LiPF_6_ salt in the presence of water (300 ppm) in the carrier gas by thermogravimetric analysis (TGA) and on-line Fourier transform infrared (FTIR). The results showed that pure LiPF_6_ salt is thermally stable up to 107 °C in a dry, inert atmosphere. However, the initial decomposition temperature reduced to 87 °C due to the presence of water (300 ppm) with the formation of POF_3_ and HF. Furthermore, the reaction rates between LiPF_6_ and water in different solvents for Li-ion batteries are in inverse proportion to the order of their dielectric constants [8]. PF_5_ can also react with water to form HF, which can react with organic solvent and the SEI layer. In addition, D. Aurbach [9] proposed that the surface chemistry can control the electrochemical behavior of both lithiated carbon anodes and lithiated transition metal electrodes. During cycling and storage, the spontaneous surface reactions between the SEI and acidic species, such as HF and PF_5_, are enhanced. That results in an increase in the electrodes’ impedance and causes capacity-fading at elevated temperatures [10,11,12]. Therefore, some reactive additives or new salts are introduced to the electrolyte to weaken the detrimental effect of decomposition of LiPF_6_, such as Vinylene carbonate (VC) [5], Li disalicilato-borate salt and silica [12]. Experimentally, Lee et al. [5] found that the SEI layer induced from VC is quite stable at elevated temperatures. However, the thermal decomposition of LiPF_6_ salt is unavoidable despite the addition of VC. In addition, silica [12] and the graphite coating [13] can absorb HF to protect the material surface from corrosion. Nevertheless, there are still some residual acidic substances to corrode the material surface.

Many researchers have focused on the anode/cathode materials, electrolytes, and separators. However, few studies have been made on current collectors, especially the anode current collector, Cu foil. Myung [14] and Shu [15] et al. found that the presence of HF in the electrolyte was essential in the formation of the metal fluoride layer on the oxide layer of the SEI and water is very important for the formation of passive layers on the surface of the copper current collector. Due to the limitations of these experiments, the microscopic mechanism of the corrosion processes on the surface of Cu foil is not clarified. Hence, combined with the existing experimental phenomena and data, the reaction mechanism of the three vital contaminants, H_2_O, HF, and PF_5_ on the Cu surface, was investigated by the first-principles method based on the density functional theory.

## 2. Computational Method and Models

**Figure 1:**
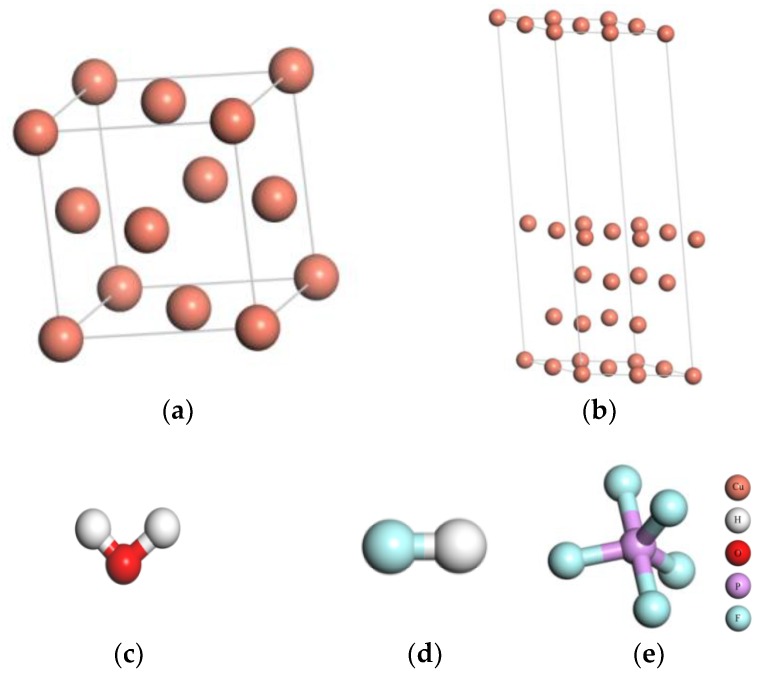
The models of Cu and three kinds of impurities: (**a**) the unit cell, (**b**) the surface periods slab model of the unit cell, (**c**) H_2_O, (**d**) HF, and (**e**) PF_5_.

The copper crystal is a face-centered cubic (FCC) structure. Space group is FM-3M. The unit cell contains 4 atoms, as shown in Figure 1a. After structure optimization, the copper lattice parameter is 3.68 Å, which is consistent with the experiment value (3.61 Å) [16]. The Cu(111) surface is the most closely packed plane which is the most stable surface. The close-packed (111) surface energy is the lowest [17]. Therefore, the Cu(111) surface was chosen to study. Considering the boundary effect, the clean Cu(111) (2 × 2) surface model with four layer slabs is constructed. Each layer contains 4 atoms, and the vacuum space is set as 15 Å, as shown in Figure 1b.

In the structural optimization of the constructed models, the bottom layer is fixed to simulate a bulk environment and the others are relaxed. The periodic model calculations are performed using the Dmol^3^ package based on the density functional theory (DFT). This is done by adopting the generalized gradient approximation [18] (GGA) with the Perdew–Wang 91 (PW91) functional [19] as implemented. All-electron Kohn–Sham wave functions are expanded in a Double Numerical basis [20,21] (DND). Brillouin zone integration is performed using Monkhorst–Pack special k-point grids. In order to get the final structure with minimum total energy, the self-consistent field cycle convergence tolerance is 1 × 10^−5^ eV and the convergence criteria of optimization is ≤2.0 × 10^−5^ Ha (1 Ha = 27.2114 eV), 0.005 Ha/Å, and 0.005 Å for energy, force, and displacement, respectively. HF, H_2_O, and PF_5_ molecules are placed on a clean surface. To calculate the dissociation energy barriers of HF on the clean and preadsorbed O atom Cu(111) surface, the transition state (TS) [22] of the surface transformation was located on the potential energy hypersurface by performing a linear synchronous transit (LST) calculation, combined with a quadratic synchronous transit (QST) calculation [23], and conjugated gradient refinements. Meanwhile, the smearing energy is set as 0.005 Ha to achieve the fast energy convergence.

## 3. Results and Discussion

### 3.1. HF, H_2_O, and PF_5_ Adsorbed on Clean Cu(111)

Many researchers studied the possible reactions of LiPF_6_ or water in organic solvents and the following equations have been accepted [24,25]:

LiPF_6_⇔LiF + PF_5_ (1)

PF_5_ + H_2_O→POF_3_ + 2HF (2)

Nonionized LiPF_6_ dissociates to PF_5_, a strong Lewis acid, and LiF in organic solvents. PF_5_ reacts with the trace amount of water in the electrolyte and generates POF_3_ and HF. PF_5_ is highly reactive and sometimes acts as catalyst [6]. Besides that, Zhao et al. [26] experimentally found that even small amounts of impurities, such as H_2_O or HF, enhanced the oxidation rate of copper considerably. Thus, the adsorption of H_2_O, HF, and PF_5_ on the Cu(111) surface are studied in this work.

**Figure 2:**
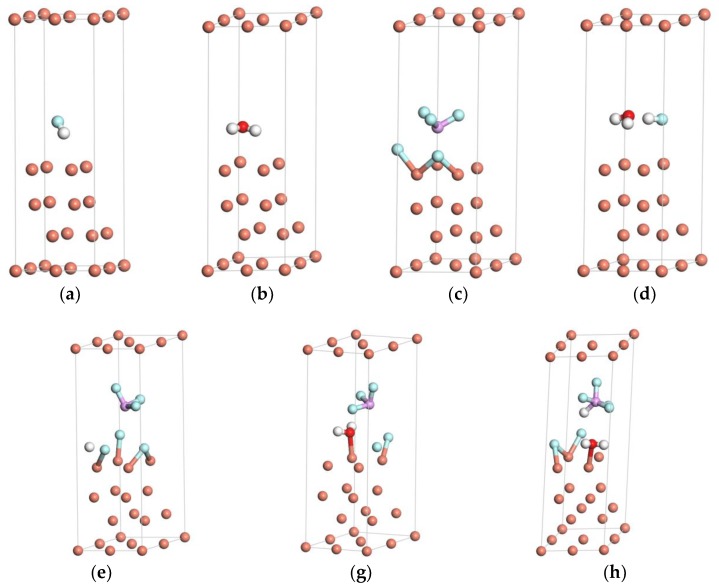
Schematic drawings of HF, H_2_O, and PF_5_ adsorbed on the Cu(111) surface after structure optimization: (**a**) HF, (**b**) H_2_O, (**c**) PF_5_, (**d**) HF and H_2_O, (**e**) HF and PF_5_, (**f**) H_2_O and PF_5_, and (**g**) HF, H_2_O, and PF_5_.

Figure 2 provides the schematic drawings of HF, H_2_O, and PF_5_ adsorbed on the Cu(111) surface after structure optimization. As shown in Figure 2a,b, the equilibrium adsorbate–substrate distances of F–Cu and O–Cu are 3.06 Å and 2.46 Å, respectively, which are evidently larger than the sum of the ionic radius of F^−^–Cu^2+^ (2.06 Å) and O^2−^–Cu^2+^ (2.13 Å). That is, the isolated HF or H_2_O will not be inclined to adsorb on the surface of Cu(111). According to Figure 2e,f, the equilibrium adsorbate–substrate distances of F–Cu and O–Cu decrease to 2.15 Å and 2.107 Å, respectively, as PF_5_ exists simultaneously with HF or H_2_O, indicating that PF_5_ can promote the adsorption of HF or H_2_O.

To further clarify the interaction between HF, H_2_O, and PF_5_, the geometrical parameters of HF and H_2_O with the stable configurations after structure optimization are calculated. Table 1 lists the bond lengths of H–F on the Cu(111) surface in Figure 2a,d,e,g. The bond length of H–F increases from 0.956 Å to 1.009 Å with the coexistence of H_2_O and HF, and it increases to 1.345 Å with the existence of HF and PF_5_. As PF_5_, H_2_O, and HF exist simultaneously, the bond length of H–F increases to 2.547 Å. Therefore, both H_2_O and PF_5_ can promote the dissociation of the H–F bond on the surface of Cu(111). Comparatively, the promotion effect of PF_5_ is more pronounced than H_2_O.

To clarify the effects of HF, PF_5_ on the adsorption of H_2_O, the bond lengths and bond angles of water on the Cu(111) surface are calculated from Figure 2b,d,f,g, which are listed in Table 2. It can be found that the H–O–H internal angle slightly increases from 103.199° to 104.939° with the simultaneous adsorption of H_2_O and HF. Meanwhile, the internal angle increases to 105.707° with the extra addition of PF_5_. No evident change was observed for the configuration of H_2_O molecules, showing that the adsorption of H_2_O is physical adsorption, which is consistent with the results illustrated by Chen et al. [27].

**Figure 3:**
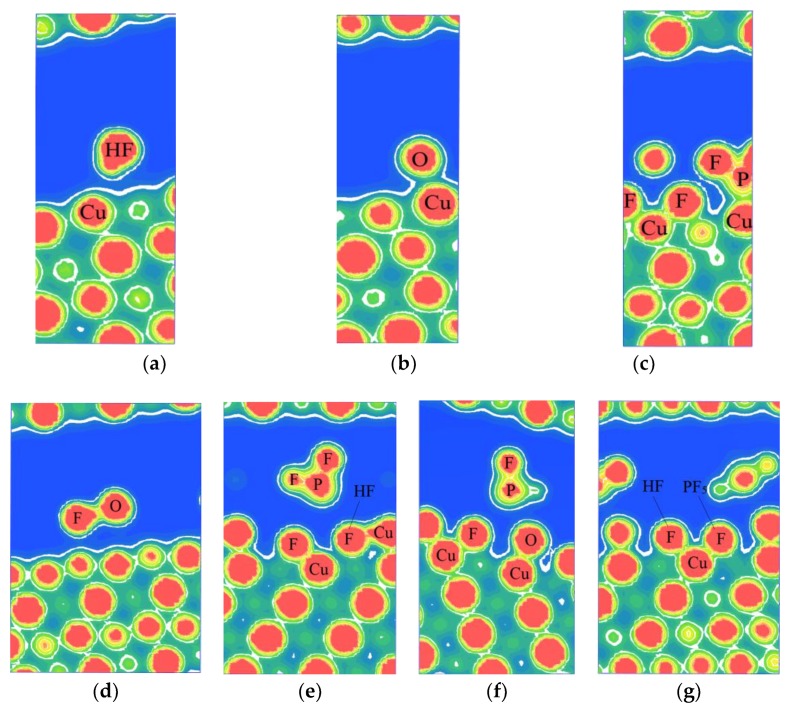
The electron density plots of the stable configurations of HF, H_2_O, and PF_5_ adsorbed on Cu(111) surface: (**a**) HF, (**b**) H_2_O, (**c**) PF_5_, (**d**) HF and H_2_O, (**e**) HF and PF_5_, (**f**) H_2_O and PF_5_, and (**g**) HF, H_2_O, and PF_5_.

Figure 3 shows the electron density plots of HF, H_2_O, and PF_5_ adsorption on the stable configurations of the Cu(111) surface. As illustrated in Figure 3a,b,d, when HF, H_2_O, or both of them exist on the Cu(111) surface, there is no obvious overlapping of electron cloud between HF or H_2_O and the surface atoms of Cu(111), suggesting that no adsorption occurs for H_2_O or HF on the surface of copper. However, obvious overlapping of electron cloud could be observed between F atoms from HF and Cu atoms as PF_5_ and H_2_O exist simultaneously, as shown in Figure 3e. The similar results could be observed in Figure 3f, as H_2_O and PF_5_ coexist. Moreover, the electron contours of F atoms and Cu atoms are smooth and round, indicating that the ionic bond forms between the F atom and Cu atom. It proves that the adsorption of HF is chemisorption. Thus, it can be concluded that PF_5_ can promote the dissociation of HF and the physical adsorption of H_2_O on Cu(111) surface. Shu at el. [15] observed P, F, and O elements on the surface of Cu foil which was immersed in the electrolyte of lithium-ion batteries for 30 days and deduced that small amounts of decomposition product (such as PF_5_) during storage of lithium-ion batteries may have vital effects on the stability of copper. Additionally, some researchers deduced that small amounts of decomposition product (such as PF_5_) during storage of lithium-ion batteries may have vital effects on the stability of copper [6].

**Figure 4:**
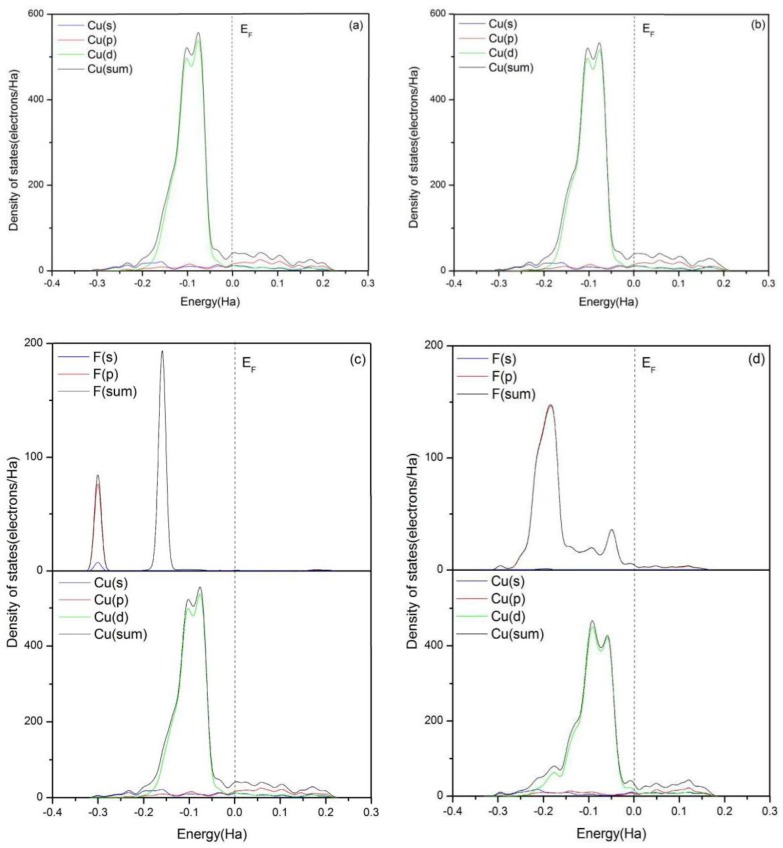

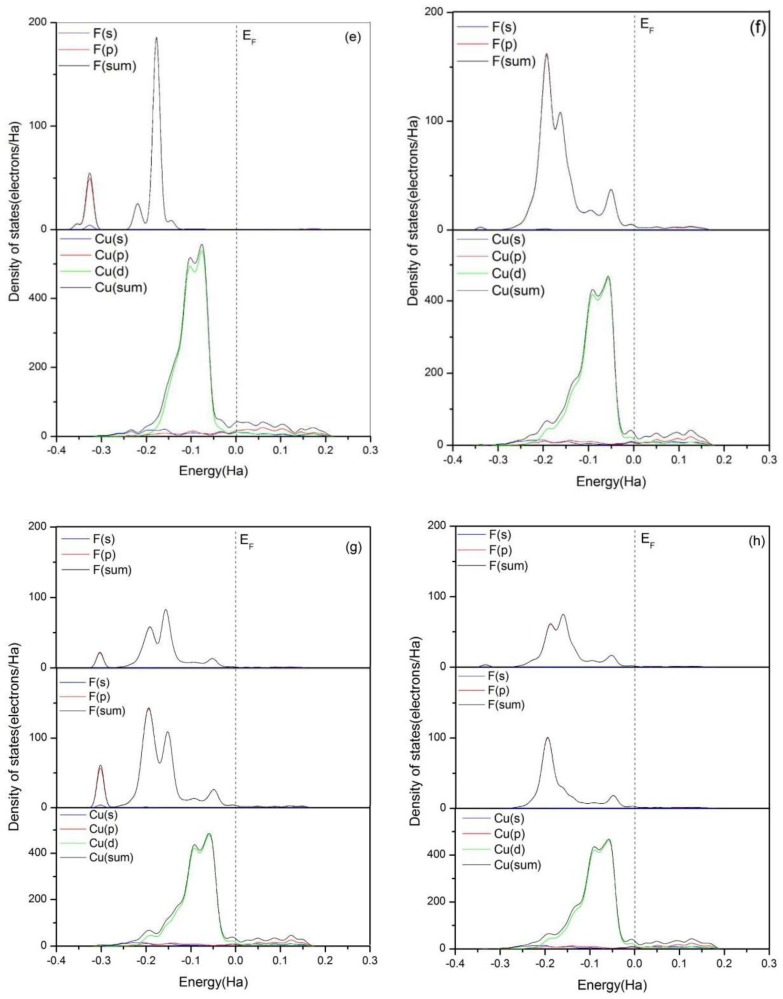
Total and partial density of states of F atom in HF, F atoms in PF_5_, which form bonds with Cu atoms, and Cu atoms in the topmost layer of clean Cu(111) surface (when the two kinds of F atoms exist together, the F from HF is above). (**a**) Clean Cu(111) surface, (**b**) H_2_O, (**c**) HF, (**d**) PF_5_, (**e**) HF and H_2_O, (**f**) H_2_O and PF_5_, (**g**) HF and PF_5_, and (**h**) HF, H_2_O, and PF_5_.

To obtain more information about the interaction between the adsorbates and the surface of Cu(111), the total and partial densities of states (DOS) of related F and Cu atoms are calculated as shown in Figure 4. The Fermi level (*E*_F_) is set as zero and used as a reference. From Figure 4a, the bonding peaks of clean Cu(111) are mainly located at the energy range between *E*_F_ and −3 Ha. The bonding electrons between −0.2 and 0 Ha are mainly dominated by the valence electrons of Cu d orbit.

As shown in Figure 4b–e, the total and partial densities of states of Cu(111) with the adsorption of HF, H_2_O, or both of them are almost identical to the clean Cu(111), which indicates there is no bond formation between HF or H_2_O and Cu(111). The results are consistent with those mentioned above. However, a new bonding peak of Cu atoms appears at the low-energy region between −0.2 and −0.15 Ha with addition of PF_5_, as shown in Figure 4d,f–h. The peak is stemming from the interaction of Cu 3d orbit and F 2p orbit. The partial densities of states (PDOS) analysis indicates the bond between F and Cu atom is ionic.

According to Figure 4g, the extra addition of PF_5_ also results in the expansion of the peak of DOS of F atom between −0.25 and 0 Ha. Meanwhile, a new bonding peak around the energy level of −0.05 Ha appears, which is related to a new hybrid orbit which is generated between Cu atom and F atoms from HF. From Figure 4h, it can be seen that the bonding peaks of F atoms around the energy level of −0.3 Ha almost entirely disappear with the existence of H_2_O, HF, and PF_5_, and DOS of F atoms is dominated by F 2p orbit. The overlapping of the hybrid orbits is enhanced and the interaction between F and Cu atoms is strengthened. Thus, it can be concluded that the trace amount of H_2_O in the electrolyte can promote the spontaneous dissociation of HF and PF_5_ on the clean Cu(111) surface.

### 3.2. The Product Formed by F and Cu Atoms

**Figure 5:**
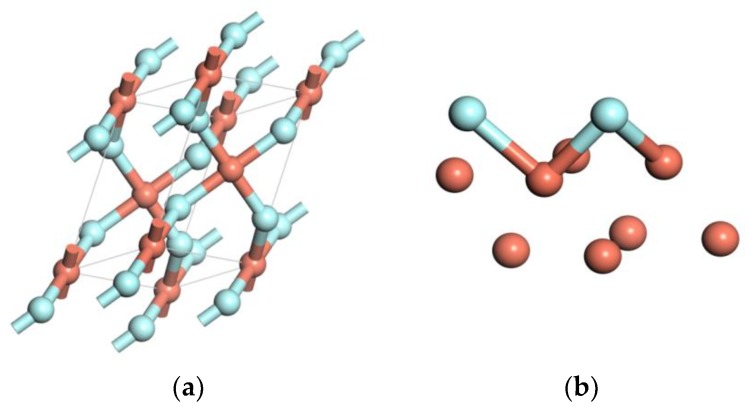
Schematic drawing: (**a**) the CuF_2_ unit cell and (**b**) the bonds formed by the F atoms and Cu atoms in Figure 2g.

Our previous work shows that CuF_2_ forms on the surface of copper after immersion in the electrolyte of lithium-ions batteries for 30 days [28]. Myung [14] also reported that metal fluoride was observed in the outer corrosion production layer. According to the lattice parameters of Fischer et al. [29], CuF_2_ unit cell is constructed to compare with the simulation results. Figure 5a presents the CuF_2_ unit cell after structure optimization. It can be found that Cu–F bond lengths of CuF_2_ are 1.929 Å and 1.946 Å, respectively, and the bond internal angle of F–Cu–F is 90.2°. Figure 5b shows the schematic diagram of the bonds formed by the F atoms and Cu atoms as illustrated in Figure 2g. The lengths of Cu–F bonds are 2.103 Å and 2.212 Å and the bond internal angle of F–Cu–F is 89.8°. The simulation results are consistent with the lattice parameter of the CuF_2_ molecule. Hence, the results demonstrate theoretically that the reaction product formed by the F and Cu atoms is CuF_2_.

### 3.3. HF Dissociation on Clean and Preadsorbed O/Cu(111) Surfaces

**Figure 6:**
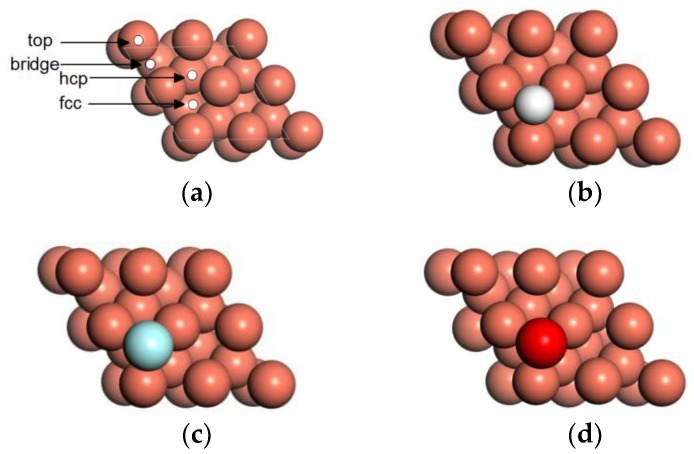
Stable adsorption configuration of adatom on Cu(111) surface: (**a**) surface adsorption sites of Cu(111), (**b**) H, (**c**) F, and (**d**) O.

In fact, Shu et al. [15] found that HF etches the copper oxides layer on the copper current collector more severely. To verify this, the dissociation energy barriers of HF on the clean and preadsorbed O/Cu(111) surfaces were calculated. During HF dissociation processes, the most stable adsorption sites of the related atoms should be provided. There are four adsorption sites of H, F, and O atoms adsorbed on the surface, and the adsorption energies are calculated as follows:

(3) $$E_{ad}=E_{a/\mathrm{Cu}\left(111\right)}-E_{\mathrm{Cu}\left(111\right)}-E_{a}$$

where $E_{\mathrm{Cu}\left(111\right)}$ is the total energy of the clean Cu(111) surface, $E_{a}$ is the total energy of the adsorbed atom, and $E_{a/\mathrm{Cu}\left(111\right)}$ is the total energy of the Cu(111) surface with the adsorbed atom. The greater the absolute value of the adsorption energy is, the stronger the interaction between the adsorbed atom and the surface. Table 3 lists the adsorption energies of H, F, and O atoms on clean Cu(111). After structure optimization, it is found that the H, F, and O atoms at the top site are relaxed to the fcc site, and the H, F, and O atoms at the bridge site are relaxed to the hcp site. According to the adsorption energies and the positions of H, F, and O atoms in Figure 6, it is concluded that the fcc site is the preferable adsorption site of H, F, and O atoms on the Cu(111) surface.

**Figure 7:**
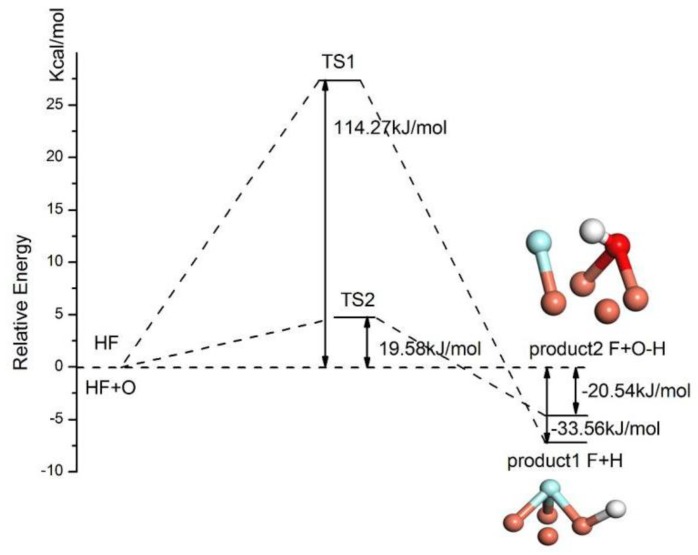
The dissociation pathway for HF on different Cu(111) surfaces.

According to the calculated results of HF adsorption configurations on the Cu(111) surface, no spontaneous dissociation of HF can be observed. Hence, the activation energy is required for the dissociation reaction. The stable adsorption configurations of HF on two surfaces are chosen as the reactants of dissociation and the stable adsorption configurations of separate H and F at two fcc sites are predicted as the products of HF dissociation. The activation barrier of the dissociation reaction is calculated by locating the transition state with the linear synchronous transit (LST) method. The energy and structural evolution of the systems for HF dissociation on clean and preadsorbed O atom on the Cu(111) surface are shown in Figure 7. It can be seen that the sites of separate H and F adsorbed on the Cu(111) surface are in agreement with that of predicted dissociation product. The energy barrier for the dissociation of HF on the clean Cu(111) surface is 114.27 kJ/mol and it reduces significantly to 19.58 kJ/mol for HF on O atom preadsorbed Cu(111) surface. Hence, the preadsorbed O atom acts as a catalyst and dramatically slashes the required activation energy of the HF dissociation.

The adsorption energies and geometrical parameters of the HF molecule on clean and O preadsorbed Cu(111) surfaces are calculated to explain the reduction of the dissociation energy barrier. From Table 4, it can be seen that the adsorption energy of HF on O/Cu(111) surfaces is 0.52 eV, which is evidently higher than that on clean Cu(111) surfaces (i.e., 0.27 eV), showing that the adsorption of HF on O atom preadsorbed Cu (111) surfaces is more stable than that on clean Cu(111) surfaces. Moreover, the bond length of HF on Cu(111) surfaces with the preadsorbed O is 0.95 Å, which is longer than that on clean Cu(111) surfaces. Thus, it can be concluded that the preadsorbed O atom can promote the break of the H–F bond. Since O atom is more electronegative than F atom, it is apt to break the H–F bond. Thus, it also demonstrates that HF is prone to etching the copper oxides on the surface of copper, as reported in the reference [14,15].

## 4. Conclusions

In lithium-ions batteries, PF_5_ stemming from the thermal decomposition of electrolytes can react with the residual water in the electrolyte and produce HF. To investigate the effects of PF_5_, H_2_O, and HF on the stability of the copper current collector of lithium-ions batteries, the adsorption of HF, H_2_O, and PF_5_ on Cu(111) surfaces was systematically studied based on the density functional theory.

(1) Both H_2_O and PF_5_ can promote the dissociation of HF. PF_5_ also has a promotion effect on the physical adsorption of H_2_O on Cu(111) surfaces. Meanwhile, the spans of the DOS of F atom from HF between −0.25 and 0 Ha enlarge obviously and a new bonding peak around the energy level of −0.05 Ha appears with the addition of PF_5_, indicating a new hybrid orbit is generated between the F atom from HF and the Cu atom. The bond between the F atom and Cu atom is ionic, and the reaction product is a CuF_2_ molecule.

(2) The most stable adsorption sites of the related atoms (H, O, and F) are all the fcc sites. The dissociation energy barrier on O adsorbate-adsorbed Cu(111) surfaces is much less than that on the clean Cu(111) surface. The preadsorbed O atom plays a catalytic role to dramatically slash the required activation energy of the dissociation of HF.

## Figures and Tables

**Table 1:** Geometrical parameters of HF molecule on Cu (111) surface in Figure 2.

Condition	(a)	(d)	(e)	(g)
d_H__–F_/Å	0.956	1.009	1.345	2.547

**Table 2:** Geometrical parameters of H_2_O molecule on Cu (111) surface in Figure 2.

Condition	(b)	(d)	(f)	(g)
d_O__–H_/Å	0.985	0.985	0.992	0.987
∠(HOH)/(°)	103.199	104.939	105.707	105.255

**Table 3:** Adsorption energies of H, F, and O atoms on Cu(111) surface (eV).

Site	Top	Bridge	Hcp	Fcc
H	−2.619	−2.617	−2.620	−2.622
O	−4.970	−4.846	−4.845	−4.971
F	−4.024	−4.001	−4.001	−4.025

**Table 4:** Geometrical parameters and adsorption energies of HF molecule on clean and O atom preadsorbed Cu(111) surfaces.

Surface Type	*E_ad_* (eV)	d_H-F_ (Å)
Clean Cu(111)	0.27	0.95
O preadsorbed Cu(111)	0.52	0.98
